# The NEUBIAS Gateway: a hub for bioimage analysis methods and materials

**DOI:** 10.12688/f1000research.24759.1

**Published:** 2020-06-16

**Authors:** Beth A. Cimini, Simon F Nørrelykke, Marion Louveaux, Nataša Sladoje, Perrine Paul-Gilloteaux, Julien Colombelli, Kota Miura

**Affiliations:** 1 Imaging Platform, Broad Institute of Harvard and MIT, Cambridge, MA, USA; 2 Image and Data Analysis group, ScopeM, ETH Zürich, Zürich, Switzerland; 3 Bioimage Analysis Hub, Institut Pasteur, Paris, France; 4 Centre for Image Analysis, Dept. of Information Technology, University of Uppsala, Uppsala, Sweden; 5 MicroPICell facility, SFR-Santé, INSERM, CNRS, Université de Nantes, CHU Nantes, Nantes, France; 6 Advanced Digital Microscopy, Institute for Research in Biomedicine (IRB Barcelona), Barcelona Institute of Science and Technology (BIST), Barcelona, Spain; 7 Nikon Imaging Center, University of Heidelberg, Heidelberg, Germany

**Keywords:** Bioimage analysis, bioimage analysts, image data, workflows, measurements, life science, training, computational methods, FAIR, reproducibility, open source

## Abstract

We introduce the NEUBIAS Gateway, a new platform for publishing materials related to bioimage analysis, an interdisciplinary field bridging computer science and life sciences. This emerging field has been lacking a central place to share the efforts of the growing group of scientists addressing biological questions using image data. The Gateway welcomes a wide range of publication formats including articles, reviews, reports and training materials. We hope the Gateway further supports this important field to grow and helps more biologists and computational scientists learn about and contribute to these efforts.

Not so long ago, microscopy images in life sciences were exclusively used as a qualitative way to present the evidence of what researchers observed under microscopes. The majority of images that appeared in papers were direct outputs of analog cameras, sometimes with enhancements using basic image processing techniques. Colocalization of proteins, as simply shown by overlaying two channels of color images, was considered a sufficiently convincing result for publication. Such ways of using image data are now insufficient: without appropriate quantitative evaluation of image data, results are no longer considered scientifically convincing. The updated way in which we interact with image data naturally calls for a more detailed description of image analysis methods. However, description of these methods, when described at all, is often relegated to the supplemental material of a scientific publication, making it more likely that *improper* analysis workflows will slip through and obscuring the importance that these techniques hold in creating verifiable scientific data.

Why isn’t the publication of image analysis methods in life science more valued? There are multiple reasons beyond simply the rapid changes in the way we use image data. Firstly, bioimage analysis workflows, each of which are developed in order to solve specific biological questions, tend to be “bespoke” and difficult to translate directly to other problems. To publish such workflows in computational or informatics journals as a stand-alone article, one would typically need to convert that workflow into a tool that is more generally applicable to a wider range of problems, or adjust the article to attempt to convince the reviewers and readers that it is a general solution. Secondly, finding an appropriate journal that accepts bioimage analysis workflows addressing a specific biological problem is a challenge. While those workflows might not be technologically novel, or might not rely on newly developed image analysis algorithms nor yield any gain in processing speed, they have a high scientific value both in quantifying parameters of biological systems and in illustrating the specific approach taken in structuring the analysis. We believe it is of utmost importance to convey to the life science community better standards to report on methods that solve a biological problem through image analysis, and to support and share the uniqueness of those solutions.

NEUBIAS has formed the community of *bioimage analysts*, who are specifically working on solving biological problems by employing their computational skills and the now-rich set of tools created for image analysis. This community needs a place to share the efforts and scientific outputs of individual analysts, and to enhance the exchange of knowledge and skills so as to increase the level of analysis for all biological scientists. The aim of the NEUBIAS gateway is to fill this gap in the publishing space and to build such a platform for sharing knowledge in bioimage analysis, further establishing the field of bioimage analysis (for further discussion, see Miura & Tosi, 2016).

Life scientists have been quite successful in increasing our understanding of how biological systems work. Simultaneously, the fields of computer vision and automated object detection and recognition have made huge strides in recognizing and interpreting images by computational algorithms, to the point that such algorithms can typically perform at near the same accuracy as their human designers, but at many times the speed and with higher consistency. Image analysis in life sciences bridges these two powerful fields and is pushing these successes of each even further, but the large community effort required to build a strong “bridge” comes from understandable differences in the practical focuses of each field. While computational scientists are constantly defining new algorithms and networks, the largest datasets, their most common applications, and many of the funding opportunities are driven by “natural images” (such as photographs), which often have different parameters than biological images (such as typically using only the RGB color space, or assuming three-dimensional images are scenes that have a “floor” and a “ceiling”). While many life science tasks also require the same kinds of object detection and classification methods as natural images, there are also needs for easily-human-interpretable measurements (such as size, shape, intensity) of the physical properties of biological systems, along with estimates of their accuracy and precision. Biological images provide other challenges less-often faced in natural images (such as custom file formats and color spaces), and may also require specialized knowledge such as pathology training to distinguish between “normal” vs “abnormal” presentations of a given object or image. Bioimage analysts attempt to span these gaps, utilizing the computational resources that have been developed and adding their own biological knowledge and expertise to create adaptations of these tools that can illuminate mechanisms and connections in biological systems.

Due to the lack of a natural home for materials that fall between fields, many valuable resources such as workflows, posters, etc., are often shown and used only once and then locked away to never be seen again. This gateway will focus on all such materials that enrich the unique and exciting interdisciplinary field of bioimage analysis. For this reason, our gateway welcomes any type of resource that contributes to the improved quantitative measurement of image data. Any aspect of bioimage analysis is welcome, as long as it improves quantifiability of biological systems. Tools discussed may be standalone programs, plugins or extensions of other pre-existing tools, or workflows that combine many pre-existing tools in a unique or informative way. Materials submitted should attempt to address the scope of the problem to be solved, the uniqueness of the solution presented, how success and failure were quantified, the reusability/generality of the solution, and its limitations. By keeping the scope of the gateway to *the improved measurement of biological systems based on image data* (be that from advancements in preprocessing, better object detection/segmentation, improved ways to measure images and/or objects, novel ways to utilize measurements, updated benchmarks of existing methods, or improved accessibility of existing methods to new audiences), we believe that we can further establish the field of bioimage analysis.

Within this gateway, image analysis methods and workflows which otherwise may have been hidden behind the “Main Text” of life science publications can now have their own spotlight. We strongly encourage all submissions to follow the FAIR principles ( Wilkinson *et al*., 2016): tools, workflows, and training materials should all be Findable, Accessible, Interoperable, and Reusable. As bioimage analysis resources typically are comprised of a combination of image data and revision-controlled code, a single online gateway, where direct links to those materials can be placed is also the ideal place for the publication of bioimage analysis works. Submitted articles and their supporting materials will be peer-reviewed by bioimage analysts, to ensure quality and appropriateness, but reviewers will not judge submissions on the basis of the magnitude of the biological discovery (if any) they were used to make. We hope this gateway helps foster true scientific discussions and information exchanges for a better and creative image analysis in life sciences.

## Data availability

No data are associated with this article.

## References

[ref-1] Miura & Tosi: “Introduction.”In *Bioimage Data Analysis*, edited by Kota Miura, Weinheim: Wiley-VCH,2016;1–3. Reference Source

[ref-2] Wilkinson MD Dumontier M Aalbersberg IJ: The FAIR Guiding Principles for scientific data management and stewardship. *Sci Data.* 2016;3:160018. 10.1038/sdata.2016.18 26978244 PMC4792175

